# Correction to LncRNA TPT1‐AS1 promotes tumorigenesis and metastasis in epithelial ovarian cancer by inducing TPT1 expression

**DOI:** 10.1111/cas.16041

**Published:** 2023-12-12

Wu W, Gao H, Li X, et al. LncRNA TPT1‐AS1 promotes tumorigenesis and metastasis in epithelial ovarian cancer by inducing TPT1 expression. *Cancer Sci*. 2019;110:1587‐1598.

By carefully comparing the original experimental pictures, the author found that for Figure 3B, the migration image of SKOV3 LV‐TPT1‐AS1 was misselected, and the correct image/Figure 3B is shown here.

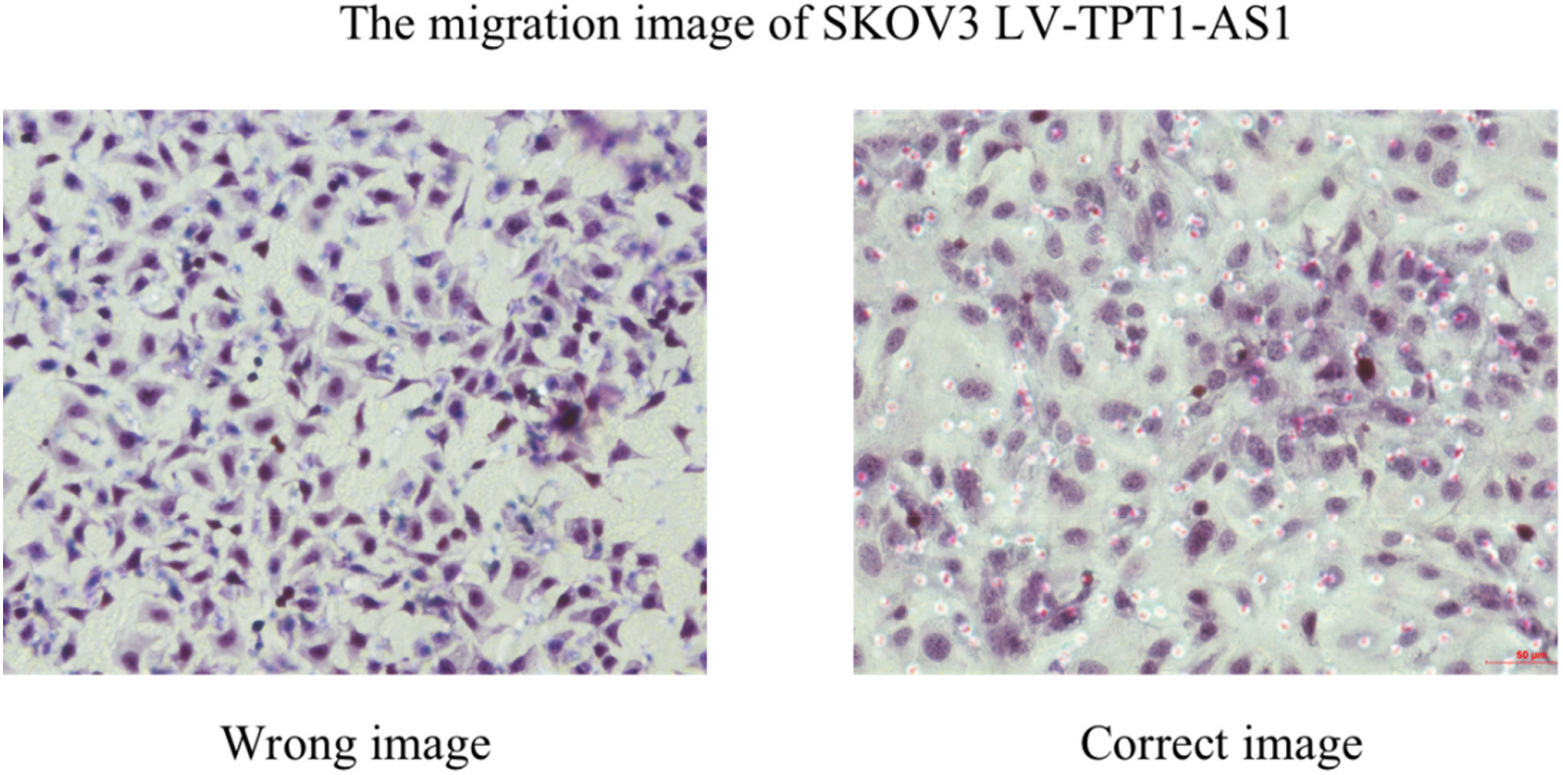

For Figure 5C, the adhesion images of SKOV3 LV‐TPT1‐AS1 + siRNA‐TPT1 were misselected. For Figure 5D, the migration image of ES‐2 LV‐TPT1‐AS1 + siRNA‐TPT1 and invasion image of SKOV3 LV‐TPT1‐AS1 + siRNA‐CON were misused. The correct image is shown here.

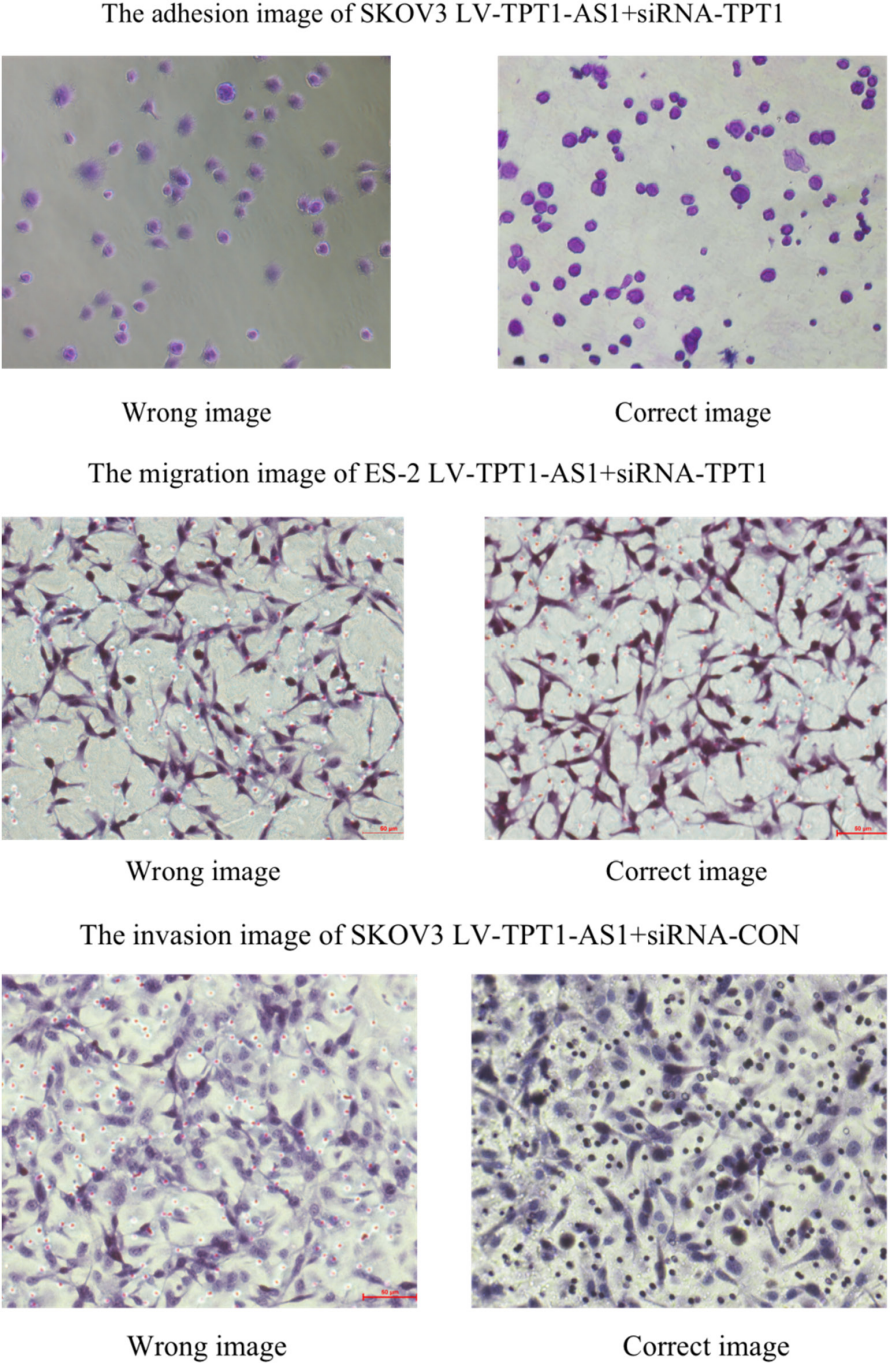

Also, by carefully comparing the original experimental pictures, the author found that for Figure 5E, the TPT1 band of SKOV3 LV‐CON+siRNA‐TPT1 was misselected, and the correct image is shown here.

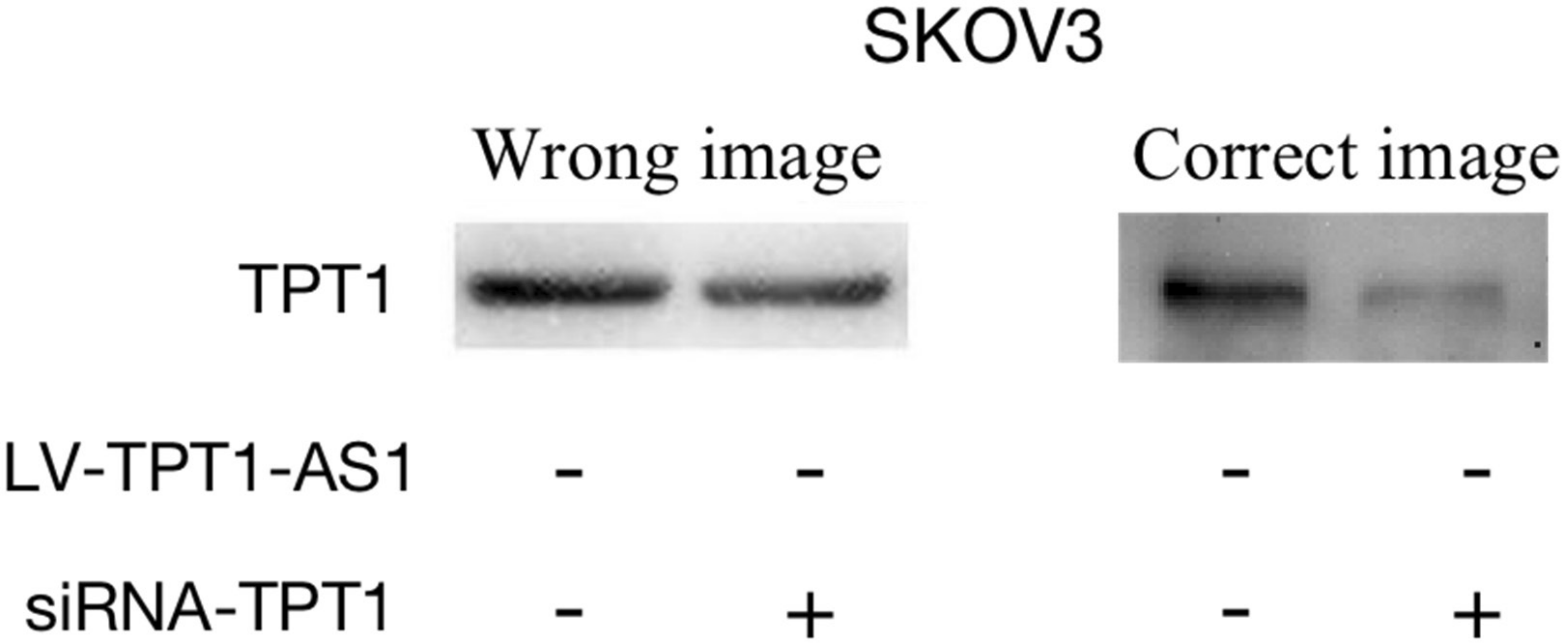

We apologize for this error.

